# Effects of standard measures in pools safety and safe-making strategies 

**Published:** 2019-07

**Authors:** Mahdi Badvi

**Affiliations:** ^*a*^Applied Research Office, Guilan Commander of Law Enforcement Force, Rasht, Iran.

## Abstract

**Background:**

Considering that pools should work under severe health and safety conditions at all hours of day and throughout year, the design of swimming pools should be in accordance with the standards of building and environmental engineering. The safety of sports places deals with how to use sports facilities to develop all aspects of sport so that it is consistent to the international standards. In most advanced countries, it has been working on safety and health standards. Unfortunately, this issue has not been taken seriously in Iran.

**Methods:**

This is a descriptive-comparative study that aims to describe the safety status of pools in the country and to examine the quality of the safety of different spaces, especially the main parts of existing pools in Iran, and the degree of inconsistency to the international standards.

**Results:**

Paying attention to the standards and safety guidelines in swimming pools can help reduce related accidents. Determining standards or desirable models is one of the most important steps in the control process.

**Conclusions:**

Examining case studies showed Iranian pools do not have the required safety features. Providing and observing guidelines for designing in the implementation and construction of pools can promote the pools’ quality and safety in the optimum level.

**Keywords:**

Pool, Standard, Safety

